# Please sir, I want some more: an exploration of repeat foodbank use

**DOI:** 10.1186/s12889-017-4847-x

**Published:** 2017-11-21

**Authors:** Elisabeth Garratt

**Affiliations:** Centre for Social Investigation, Nuffield College, New Road, Oxford, OX1 1NF UK

**Keywords:** Food insecurity, Food banks, Recession, Health inequalities, Nutrition, Food sufficiency, Food assistance

## Abstract

**Background:**

The sharp rise in foodbank use in Britain over the past five years suggests a proliferation of food insecurity that could herald a public health crisis. However, trends in foodbank use rely on imperfect figures that do not distinguish between single and repeat visits. Consequently, the true prevalence of foodbank use in Britain is unknown. By identifying repeat visits, this study provides the first estimate of the proportion of people using foodbanks.

**Methods:**

Using data on referrals to West Cheshire Foodbank in the UK, this study offers a case study of 7769 referrals to one foodbank between 2013 and 2015. Foodbank use was explored in descriptive statistics, then negative binomial regression models were used to identify the household characteristics associated with the number of foodbank visits.

**Results:**

Between 0.9 and 1.3% of people in West Cheshire sought assistance from West Cheshire Foodbank between 2013 and 2015. If scaled up nationally, this would equate to an estimated 850,000 people across Britain. The number of total recipients increased by 29% between 2013 and 2015, while the number of unique recipients rose by 14%. Multivariate analysis revealed that a larger number of visits were recorded in 2015 and among working-age and one-person households, while households referred due to domestic abuse and unemployment made fewer visits.

**Conclusion:**

Food insecurity has emerged as a crucial challenge facing UK health professionals and policymakers. This study provides the first estimate of the proportion of individuals receiving emergency food in a single case study location, and demonstrates that foodbank use is becoming more prevalent, although headline figures overstate the scale of this growth. The potential nutrition and wider health consequences of reliance on emergency food – especially among those using foodbanks on multiple occasions – warns of an unfolding public health crisis.

**Electronic supplementary material:**

The online version of this article (10.1186/s12889-017-4847-x) contains supplementary material, which is available to authorized users.

## Background

The proliferation of foodbank use over the past five years suggests that Britain is facing a mounting nutrition and public health crisis [1]. In 2013, the striking estimate by Oxfam and Church Action on Poverty that 500,000 people were reliant on foodbanks [2] was accompanied by an explosion of media interest in foodbank use [3]. By 2014–15, the Trussell Trust – the largest supplier of emergency food in Britain, and the only such organisation to record the number of people receiving emergency food – reported that this figure had exceeded one million people [4], a level that has persisted in subsequent years. Recent figures estimated that 21% of people in England experienced food insecurity in 2016 [5], yet food insecurity is not routinely monitored in Britain, making it impossible to determine the scale of this problem over time. Foodbank use has therefore been used to capture extreme experiences of food insecurity, and the Trussell Trust have been instrumental in establishing food insecurity and foodbank use as pressing research and policy issues, with a corresponding rise in research interest on the topic (eg: [6–8]).

While undoubtedly powerful, headline figures on foodbank use are problematic because they do not account for repeat visits. Canadian research reports high levels of repeat use [9], but differences in foodbank operations between countries means that the same patterns may not be true in Britain. The Trussell Trust operates a voucher system whereby those seeking assistance must be referred by a frontline care professional. This system aims to ensure that emergency food is only distributed to those in genuine need, while attempting to counter critics’ arguments that people simply take advantage of a free good [10]. Recipients are ordinarily allowed to claim three vouchers for emergency food (with additional vouchers issued at the discretion of foodbank managers), and Trussell Trust figures do not account for these repeat visits. Consequently, Trussell Trust figures do not identify the number of individuals receiving emergency food. The proportion of people using foodbanks in Britain is therefore unknown.

One attempt to identify the number of unique recipients at Wandsworth foodbank in London reported that households made an average of 1.8 visits each year [11]. While valuable, this estimate does not relate to a defined population, making it impossible to estimate the proportion of people receiving emergency food. The scale of foodbank use in Britain therefore remains uncertain. A related unanswered question is whether demographic characteristics are associated with households’ short- and long-term foodbank use. This question has been explored in the US [12, 13] but not in Britain, leaving unknown the characteristics of extremely vulnerable people for whom foodbanks necessarily offer only a temporary form of emergency relief. This study provides the first estimate of the proportion of individuals receiving emergency food using data from West Cheshire Foodbank in north-west England between 2013 and 2015. It also explores the household characteristics associated with number of visits to offer the first available insights into repeat foodbank use.

The social gradient in dietary quality makes diet an important contributor to health inequalities [14], so the existence of food insecurity should be a concern to both healthcare professionals and policymakers. Although causality cannot be established, food insecurity is associated with poor quality [15] and nutritionally inadequate diets [16], and concerns have been raised about the quality of emergency food [17]. Alongside dietary considerations, experiences of food insecurity are also associated with increased risks of general health impairments in adults and children [18, 19], alongside a range of chronic conditions including diabetes [20, 21], overweight and obesity [22, 23], HIV [24], asthma, migraines, and heart disease [25], and risky health behaviours [26]. Experiences of food insecurity are also linked with impaired mental health in adults [27–29] and children [30, 31], alongside elevated suicide rates [32]. Likewise, foodbank use is concentrated among people with poor health and disabilities, especially mental health problems [7, 33]. The provision and suitability of food therefore contributes to persistent health inequalities. The burden of food insecurity is not only felt by individuals, as household food insecurity is associated with higher healthcare costs [34]. In a socialised healthcare system like the British NHS, these costs exert pressures on already tight government budgets. Reducing food insecurity is consequently a goal worth pursuing to both relieve some financial pressures on healthcare services, and to maximise people’s quality of life.

Practitioners are increasingly well versed in the problem of food insecurity, and may have seen its effects first-hand: one in six of 500 GPs reported having been asked to refer patients to foodbanks in 2014 [35]. Likewise, the doubling of malnutrition-related hospital admissions between 2008 and 2012 led clinicians to warn of an impending public health emergency [1]. Britain is still experiencing the repercussions of the global financial crisis [36] and considerable reforms to welfare provision, which have been demonstrated to contribute to growing food insecurity and foodbank use [37, 38]. Likewise, declining food expenditure and the erosion of nutritional quality over this period [39] and warnings of rising food prices in the wake of Britain’s vote to leave the EU [40] could serve to widen persistent health inequalities. Food insecurity and foodbank use is therefore a crucial area for preventative action to protect the nation’s health.

This study provides the first estimate of the prevalence of UK foodbank use through a case study of one foodbank. First, it separately explores trends in unique and total foodbank use to determine the average number of visits and gauge the extent to which repeat foodbank visits underpin reports of rising foodbank use in Britain. Second, it derives separate estimates of the proportion of adults and children receiving emergency food from foodbanks. This study third explores the distribution of emergency food parcels between households receiving food on different numbers of occasions. Finally, multivariate analyses are used to identify the characteristics of households who made different numbers of foodbank visits.

## Methods

### Data

Data comprised all referrals to West Cheshire Foodbank in north-west England – one of 429 projects in the Trussell Trust’s foodbank network – between 2013 and 2015. West Cheshire foodbank launched in November 2012 and supplies emergency food at six distribution centres. Households seeking emergency food must be referred by a frontline care professional who issues foodbank vouchers containing the name, address, and age group of the recipient, the number of adults and children in their household, and the nature of the crisis that led them to seek emergency food. A wide range of frontline services, including the Citizens Advice Bureau, registered social landlords, schools, and GPs may refer people to foodbanks. Vouchers are then redeemed at foodbanks in exchange for a standardised parcel of three days’ worth of non-perishable, nutritionally balanced food [41]. The information contained in vouchers is then entered and collated for the primary purpose of stock control, and the quality and consistency of these data have not been formally assessed. The Trussell Trust operates a ‘three voucher rule’ whereby recipients may claim three vouchers, after which the frontline care professional must make special arrangements with the foodbank before issuing further vouchers; foodbank managers are also authorised to issue additional vouchers. In light of rising demand [42], this rule attempts both to maintain the sustainability of a model that relies predominantly on public donations, and impose accountability on care professionals to encourage longer-term solutions [43].

Although the Trussell Trust is not the only supplier of emergency food (and this limitation is considered in the Discussion), these data have the greatest potential to estimate the number and characteristics of people receiving emergency food. As administrative data, missing data is very scarce. Missing data on date of foodbank visit (24 cases) and age (13 cases) reduced the dataset by just 0.47%, from 7806 to 7769 cases.

### Data preparation

To establish annual estimates of the number of unique and repeat recipients and to enable comparisons with annual national-level Trussell Trust data, repeat visits were defined as receiving emergency food from West Cheshire Foodbank more than once in the same calendar year. Repeat users were identified on the basis of duplicated name and address information. Individuals with the same name were considered the same person, even if their address was not consistent, as the highly mobile nature of disadvantaged groups means that a more stringent approach of requiring people to live at the same address over the year would risk missing repeat visits and consequently over-estimating the prevalence of foodbank use. Exploratory analyses revealed that nearly one in five (18.9%) of households who received more than one food parcel moved home throughout the year. Name information was manually amended to correct spelling mistakes and abbreviations where these could be unambiguously identified.

Foodbank vouchers are issued to an individual for their household, so – under the expectation food was shared within the household – food parcels distributed to people with the same surname and address were considered repeat users, even if the household’s size and composition changed over the year. Analogous to the decisions relating to address information described above, requiring household size and composition to remain stable throughout the study period would risk missing repeat visits and consequently over-estimating the prevalence of foodbank use. Households’ size and composition will fluctuate in response to dynamics including relationship breakdown and children visiting separated parents, and these dynamics are especially likely among disadvantaged groups such as foodbank users. Nearly one-third (29.2%) of repeat users saw a change in their household composition throughout the year. These decisions relating to the identification of repeat visits were made to balance the risks of over- and under-estimating the prevalence of repeat use, on the expectation that households receiving emergency assistance are likely to be both geographically mobile and have fluid household structures.

All analyses were restricted to households living in the 34 wards within the catchment of West Cheshire Foodbank, which covers the towns of Chester and Ellesmere Port and Neston. Households were identified as roofless if they provided no address on any occasion over the year; such households were assumed to reside in these wards. Nationally, homelessness accounted for 5.4% of referrals to Trussell Trust foodbanks in 2015–16, and this figure is thought to under-estimate the influence of homelessness on foodbank use [44]. Assessments for assistance are made only by trained staff who are registered signatories with the foodbank, making it unlikely that people were incorrectly identified as roofless on account of missing address information. As noted above, missing data on other fields was negligible. Households living in temporary accommodation in refuges, hostels, or with friends or family could not be reliably identified, so this measure of rooflessness identified only the most vulnerably housed (sometimes referred to as ‘literally homeless’).

Trussell Trust foodbanks collect data on reasons for referral. ‘Other’ reasons include high housing expenses, being released from prison, and benefit sanctions (accounting for 9.8% of referrals to West Cheshire Foodbank). Due to small numbers, reasons of child holiday meals, delayed wages, refused crisis loan and refused short-term benefit advance (together accounting for 2.1% of referrals to West Cheshire Foodbank) were also recoded as ‘other’. Referrals due to homelessness (5.1%) were additionally recoded as ‘other’ to avoid overlap with the rooflessness measure.

Food insecurity is associated with households’ material hardship [15, 45] and may also relate characteristics of the local area [46], including food access [47]. Foodbank vouchers contain no information on these measures, so households’ postcode data was georeferenced to the 2015 Index of Multiple Deprivation (IMD) to supply contextual information on multiple and income deprivation. Roofless households were assigned the most deprived quintile. Sensitivity analyses replicated all results when roofless households were assigned to each of the five multiple and income deprivation quintiles. The demographic characteristics of the sample are summarised in Table 1; these figures are broken down by rooflessness status in Additional file 1.

**Table 1:** Demographic characteristics of households receiving assistance from West Cheshire Foodbank in 2013

		Number of households (*n*)	Proportion of households (%)	Mean annual visits
Age group ^a^	17–24	300	14.1	2.5
	25–64	1783	83.8	3.6
	65 and older	45	2.1	2.9
Rooflessness status	Housed	2038	95.8	3.4
	Roofless	90	4.2	3.6
Household type	One person	1312	61.7	3.7
	Couple, no children	188	8.8	2.9
	Couple parent with children	363	17.1	3.1
	Lone parent with children	224	10.5	2.8
	Other ^b^ household type	41	1.9	2.5
Reason for referral	Benefit change	386	18.1	3.3
	Benefit delay	827	38.9	3.7
	Debt	191	9.0	3.3
	Domestic abuse	22	1.0	2.2
	Low income	304	14.3	3.5
	Sickness/ill health	28	1.3	2.9
	Unemployed	30	1.4	2.4
	‘Other’ ^c^ reason	340	16.0	3.1
Index of Multiple Deprivation quintile	1 (most deprived)	1449	68.1	3.4
	2	342	16.1	3.2
	3	114	5.4	3.7
	4	117	5.5	2.6
	5 (least deprived)	106	5.0	5.5
Income Deprivation quintile	1 (most deprived)	1386	65.1	3.3
	2	397	18.7	3.6
	3	106	5.0	2.7
	4	145	6.8	3.6
	5 (least deprived)	94	4.4	4.5
Total		2128	100.0	3.4

^a^ Age group relates to the person claiming the food parcel only

^b^ ‘Other’ households are those containing three or more adults, approximately half of which also contained children

^c^ Includes child holiday meals, delayed wages, refused crisis loan, refused short-term benefit advance, and homelessness

### Statistical analyses

The numbers of total, unique, and repeat households receiving emergency food from West Cheshire Foodbank are first presented graphically. Next, the proportion of the population receiving emergency food are then derived using data on the prevalence of foodbank use in combination with annual ward-level population estimates for adults and children available from the Office for National Statistics.

Negative binomial regression models are then used to explore associations between households’ characteristics and the number of foodbank visits. Negative binomial models are more suitable than Poisson models when data are over-dispersed (the variance exceeds the mean number of visits) as they are here. Data are pooled from 2013 to 2015; to account for non-independence of households, all models adjust for household-level clustering.

## Results

### What proportion of emergency food recipients received assistance more than once?

**Fig. 1:**
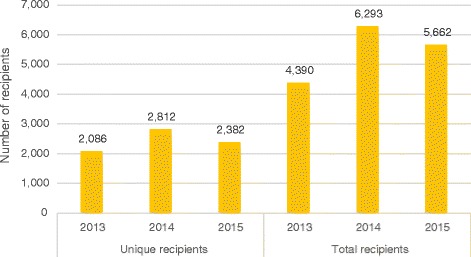
Number of unique and total recipients of emergency food in West Cheshire, 2013–2015

Figure 1 shows the number of unique and total recipients of emergency food from West Cheshire Foodbank between 2013 and 2015. Four main points are clear: first, the number of total recipients is more than twice the number of unique recipients. Headline figures from the Trussell Trust on the number of total recipients therefore over-estimate the number of unique recipients of emergency food. Second, the overall growth in the number of unique recipients between 2013 and 2015 is consistent with national rises in total foodbank use. Therefore, while national Trussell Trust data does overstate the scale of foodbank use, it is still accurate to talk of a small rise in uptake of emergency food. Third, the number of both total and unique recipients peaked in 2014 before declining slightly in 2015, suggesting that 2014 was a particularly busy year for the foodbank. Fourth, the rising number of total recipients outpaced growth in the number of unique recipients: the mean number of visits rose steadily from 2.1 in 2013 to 2.2 in 2014 and 2.3 in 2015. Between 2013 and 2015, the number of unique recipients grew by 14% while the number of total recipients increased by 29%. This pattern is replicated among adults and children [see Additional file 2]. Therefore, although the number of unique recipients grew between 2013 and 2015, repeat visits contributed disproportionately to rising foodbank use over this period.

### What proportion of people use foodbanks in West Cheshire?

**Fig. 2:**
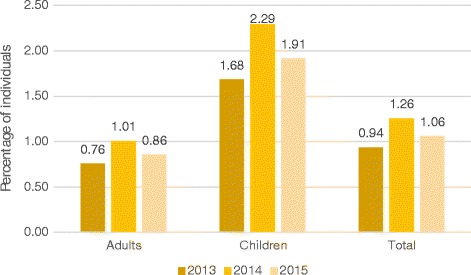
Proportion of adults and children in West Cheshire receiving emergency food, 2013–2015

The proportion of the population using foodbanks can be estimated by combining local population estimates with data on the number of unique emergency food recipients. Figure 2 shows the proportion of people in West Cheshire who received emergency food between 2013 and 2015. These proportions varied slightly over time but at its busiest in 2014, 1.01% of adults and 2.29% of children living in West Cheshire had received emergency food from West Cheshire Foodbank.

### How many food parcels are supplied to unique and repeat users?

**Fig. 3:**
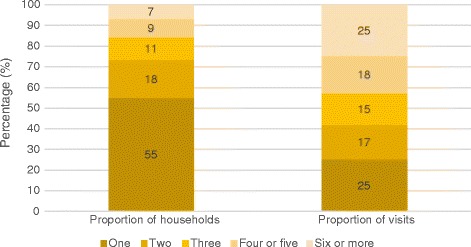
Number and composition of visits to West Cheshire Foodbank, 2013–2015

A breakdown of the number of visits made by each household, and the proportion of total food parcels received by these households between 2013 and 2015 are presented in Fig. 3. Over half of households received one food parcel, and these households accounted for one-quarter of food parcels distributed over this period. A further quarter of food parcels were received by the 7% (or 237) households who received six or more food parcels in a year. The distribution of total food parcels is therefore highly skewed and is concentrated among the small number of households who received emergency food on multiple occasions. Furthermore, the proportion of food parcels distributed to households who received emergency food on four or more occasions rose from 38% in 2013 to 47% in 2015, demonstrating that this concentration is intensifying [see Additional file 3].

### What are the characteristics of households experiencing repeat visits to foodbanks?

Table 2 presents the results of negative binomial regression models predicting households’ annual number of visits to West Cheshire Foodbank. The unit of analysis is the household, so the model estimates represent the influence of households’ characteristics at the time of their first foodbank visit on predicting their annual number of visits. The data comprise all visits to West Cheshire Foodbank, not a sample of visits, so tests of significance are not strictly necessary. Significance tests are nonetheless reported to indicate the level of confidence in the reported statistical associations between variables. Model coefficients are reported in terms of incidence rate ratios, compared with the reference value. For example, in the adjusted multivariate model the mean number of visits was 12.3% higher in 2015 than in the base year of 2013.

**Table 2:** Negative binomial regression results exploring visits to West Cheshire Foodbank by household characteristics, 2013–2015

		Number of visits	
		Unadjusted bivariate IRR	Adjusted multivariate IRR
Year	2013	1.000	1.000
		(0)	(0)
	2014	1.053	1.045
		(0.035)	(0.034)
	2015	1.123***	1.123***
		(0.039)	(0.039)
Age	17–24	0.850***	0.855***
		(0.036)	(0.036)
	25–64	1.000	1.000
		(0)	(0)
	65 and over	0.801*	0.775**
		(0.076)	(0.075)
Roofless status	Housed	1.000	1.000
		(0)	(0)
	Roofless	1.035	1.026
		(0.087)	(0.088)
Household type	One person	1.113	1.128*
		(0.062)	(0.063)
	Couple, no children	1.000	1.000
		(0)	(0)
	Couple parent with children	0.884	0.890
		(0.057)	(0.057)
	Lone parent with children	0.993	0.993
		(0.069)	(0.068)
	Other household type	0.935	0.925
		(0.106)	(0.105)
Reason for referral	Benefit change	1.000	1.000
		(0)	(0)
	Benefit delay	1.043	1.032
		(0.047)	(0.046)
	Debt	1.090	1.088
		(0.077)	(0.075)
	Domestic abuse	0.801**	0.830*
		(0.066)	(0.071)
	Low income	1.020	1.032
		(0.057)	(0.057)
	Sickness/ill-health	1.061	1.060
		(0.146)	(0.145)
	Unemployment	0.822	0.813*
		(0.085)	(0.082)
	‘Other’ reason	0.936	0.927
		(0.048)	(0.048)

*IRR* incidence rate ratio. Standard errors in parentheses. * *p* < .05 ** *p* < .01 *** *p* < .001

The multivariate analysis reveals that the number of foodbank visits varied according to households’ characteristics. A larger number of visits were recorded in 2015 and among one-person households, while fewer visits were made by the oldest and youngest recipients, and households seeing assistance due to domestic abuse and unemployment. The numbers of visits were comparable in 2013 and 2014, and did not vary significantly between couples (with and without children), lone parents with children, and ‘other’ households. Experiences of rooflessness were not associated with the number of foodbank visits. People from all 34 catchment wards visited the foodbank, and represented the full range of income and multiple deprivation. Multiple and income deprivation displayed unclear associations with foodbank use [see Additional file 4] so were excluded from the multivariate model.

**Fig. 4:**
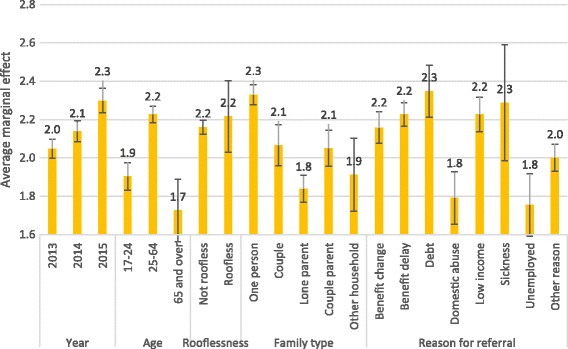
Marginal associations of household characteristics with number of visits to West Cheshire Foodbank, 2013–2015

All results are displayed graphically as marginal associations in Fig. 4, which shows the mean numbers of visits for households with each characteristic, holding constant their other characteristics. This demonstrates, for example, that the mean number of visits to West Cheshire Foodbank was higher among 25–64 year-olds (2.2 visits per year) than among recipients aged 17–24 and 65 and over (1.9 and 1.7 visits). Taken together, these analyses demonstrate that households’ number of visits varied considerably by their demographic characteristics.

## Discussion

The recent growth in foodbank use suggests that Britain could be facing an escalating nutrition and public health crisis. This study provides the first estimate of the proportion of individuals using foodbanks in West Cheshire between 2013 and 2015. Between 0.9 and 1.3% of people living in the catchment area of West Cheshire Foodbank were estimated to have received emergency food from the foodbank each year. These proportions were consistently higher for children than adults. The number of both total and unique recipients rose between 2013 and 2015, but peaked in 2014, and whether this trend reflects minor annual fluctuations or longer-term declines in foodbank use remains to be seen. Growth in the distribution of emergency food was inflated by a rising number of people visiting the foodbank on multiple occasions, indicating – as expected – that headline Trussell Trust figures overstate the scale of foodbank use in Britain. Still, the 14% rise in the number of unique recipients between 2013 and 2015 does clearly demonstrate that foodbank use is becoming more widespread.

Households’ number of foodbank visits varied considerably according to their demographic characteristics. Compared with the reference categories, a larger number of visits were recorded in 2015, and in working-age and one-person households. Working-age adults (especially those without children) have been worse affected by falling real-terms earnings following the recession [48]. Similarly, persistent poverty is concentrated among people living alone [49], consequently this group may have less protection against the income shocks that commonly trigger foodbank use [50]. Recent research also corroborates the over-representation of single men among foodbank users [33]. Households referred due to domestic abuse and unemployment made fewer visits. Consistent with past research, area-level material deprivation was not clearly associated with foodbank use [46]; experiences of rooflessness were also not associated with repeat use.

This study provides the first estimate of the proportion of people using foodbanks, so no direct comparisons with previous research are possible. Nonetheless, households in Wandsworth were estimated to receive on average 1.8 vouchers annually [11]. These figures are broadly similar with those reported here and therefore reinforce the current results. The rising number of unique recipients of emergency food in West Cheshire is also consistent with aggregate national trends [37] and trends in emergency food receipt among children [51]. This increase also strengthens broader survey evidence for growing food insecurity in Britain [52–54] and Europe [38, 55, 56].

The high prevalence of repeat foodbank use among certain groups identified in the current analyses should concern both practitioners and policy makers. Nationally, intakes of saturated fat and salt are above UK dietary recommendations, while fruit and vegetable intakes do not meet recommended intakes. Concerns about dietary quality are therefore widespread and by no means limited to low-income groups. However, alongside these widespread dietary deficiencies, there also exists a clear social gradient in dietary quality: lower-income groups typically consume less protein, fruit, and vegetables than higher-income groups [14]. People’s risk of consuming a poor-quality diet is therefore not shared equally across social groups. Food insecurity is associated with poor quality [15] and nutritionally inadequate diets [16], and although emergency food is designed to be nutritionally balanced, its quantity and quality cannot be guaranteed [17, 57], potentially intensifying the likelihood of nutritional deficiencies among foodbank users, who are already at risk of poor health and disabilities [7, 33]. The overarching role of income in determining food insecurity demonstrates the importance of policies aimed at protecting minimum wage levels and the availability of welfare benefits [38].

Practitioners also have a role to play in considering the quality of their patients’ diets. In particular, GPs who are asked to refer patients to foodbanks (approximately one in six GPs surveyed in 2014 [35]) should monitor the number of such referrals and pay particularly close attention to nutritional quality among these vulnerable patients.

If scaled up, the estimate of 1.3% of people receiving emergency food in 2014 would equate to an estimate in the order of 850,000 people across Britain. Other estimates that 21% of adults in England (nearly 11 million) in 2016 [5] and 10% of adults in Britain (8.4 million) experienced food insecurity in 2014 [58] demonstrate that estimates of foodbank use are by no means equivalent to the number of food insecure individuals. Foodbanks are commonly considered a ‘last resort’ [52, 59, 60] and research from Canada and Scotland reveals that fewer than only a minority of food insecure households use foodbanks [61–63], although this figure rises among certain marginalised groups [9]. The current figures are therefore likely to capture only extreme experiences of food insecurity. The nutritional challenges of food insecurity are therefore likely to be far greater than analyses of foodbank use would suggest.

### Study limitations and strengths

The data and methods used in this study have three potentially noteworthy limitations. First, these analyses relate only to people accessing emergency food from the Trussell Trust in a single case study location. Those receiving emergency food from independent foodbanks or other sources are not counted. The Trussell Trust collates an online directory of all their foodbanks (currently standing at 429, with 1350 distribution centres), and in May 2017, at least 651 independently run UK foodbanks were known to exist [64]. Elsewhere, a case study of one city in England similarly identified seven Trussell Trust foodbanks alongside 30 independent providers [60]. Furthermore, as a Christian charity, ethnic and religious variation among the Trussell Trust’s client base may be limited [65], so the present analyses could also understate the diversity of people receiving emergency food. The current analyses will therefore inevitably underestimate the proportion of people receiving emergency food in West Cheshire, so the scale of food insecurity and foodbank use is almost certainly higher.

Second, the data cover a geographical area that cannot fully represent the heterogeneity of Britain. In particular, West Cheshire is ethnically more homogenous than Britain overall. West Cheshire is also slightly more affluent than Britain: 7 % of small areas were in the most deprived decile of the 2015 Indices of Multiple Deprivation, while 10% fell into the least deprived two deciles. Likewise, in 2011 the area’s unemployment rate was lower than England and Wales (3.7 and 4.4%) and employment rate higher (63.2 and 61.9%) [66]. The results reported here may therefore not be replicated in other geographical areas, or in Britain overall. When considering broader estimates of foodbank use, any bias introduced by the local focus of the current analyses will therefore serve to under-estimate the prevalence of foodbank use in Britain. National estimates of foodbank use are likely to be substantially higher. Nonetheless, the characteristics of people using West Cheshire Foodbank and their reasons for referral are however comparable with national data, suggesting that the current results are applicable beyond West Cheshire [67].

Third, all analyses relied on administrative data that were designed for the purposes of stock control and never intended for research use. Consequently, the breadth and quality of these data may be lower than those of data collected specifically for research purposes. For example, no data are collected on the sex or employment status of those seeking assistance. Furthermore, the single field recording households’ reasons for referral is inevitably unlikely to fully capture the circumstances that lead to people seeking assistance from foodbanks [50]. Limited detail in the address information also meant that it was only possible to differentiate between housed and roofless households, a broad distinction that obscures variation in housing precarity and identifies only the most vulnerably housed. However, the extremely low prevalence of missing data does mean that bias is unlikely to affect the current results.

These drawbacks should be placed within the context of three key strengths. First, the analyses cover three years’ data, so capture robust temporal patterns that cannot be disregarded as statistical anomalies relating to a particular year. Second, the Trussell Trust data collection system – while imperfect for research purposes – provides information on all referrals to West Cheshire Foodbank, with no influence of sampling error and almost no missing data, so the current results are valid and lack bias. Finally, the inclusion of basic individual demographic information and the use of local area-level multiple deprivation data enabled the number of foodbank visits to be explored directly in relation to households’ characteristics, and indirectly in relation to area-level multiple deprivation.

As already recognised, the current study is necessarily limited by its focus on a specific geographical location; the current results therefore can at best suggest, not equate to, national figures. Deriving a national estimate of the proportion of people receiving emergency food would require these analyses to be repeated using a nationally-representative sample of foodbanks, a task that is not currently possible. Instead, there is a growing consensus among clinicians, researchers, and civic organisations that a national surveillance system is needed to systematically monitor food insecurity and foodbank use in Britain [37, 68, 69].

This first exploration of households’ number of visits in relation to their demographic characteristics offers insight into the characteristics of households receiving short- and longer-term assistance from foodbanks. A more detailed understanding of these different groups – especially characteristics including ethnicity, employment status and mental health that could not be assessed here – would be helpful to supplement the current results. As Trussell Trust data do not record the sex of emergency food recipients, and existing evidence on food insecurity and use of emergency food in men and women reports inconsistent results [25, 26, 34, 63, 70], further research on this topic is warranted. Qualitative research has begun to provide detailed information about the challenging lives of people receiving emergency food [7, 50, 60, 71, 72], while also serving to listen directly to a group who are mentioned frequently but seldom heard [3]. These studies are valuable in identifying the groups most at risk of food insecurity. Nonetheless, it is important that demographic differences in foodbank should not be interpreted as necessarily indicative of individual responsibility to the detriment of economic determinants of food insecurity: 23% of adults in the lowest income quintile in England reported food insecurity, compared with just 3% of those in the highest income quintile in 2016 [5]. In parallel, evidence linking welfare reform with food insecurity [38] and foodbank use [37, 63, 73] further reinforces the crucial role of material resources in determining food insecurity.

## Conclusions

The stark rise in the provision and uptake of emergency food in Britain over the past five years has firmly established food insecurity as a pressing issue facing policymakers and health professionals. This study provides the first estimate of the proportion of individuals (distinct from the number of food parcels distributed) receiving emergency food in one area of Britain. In 2014, 1.3% of people in West Cheshire received emergency food from the Trussell Trust, and this proportion was higher among children. If scaled up, this equates to an estimate in the order of 850,000 people across Britain. This figure almost certainly under-states the prevalence of foodbank use in both West Cheshire and Britain. The risks of nutritional deficiencies among food insecure individuals and the high prevalence of mental and physical health problems among people who do not eat adequate diets make ensuring food security for all an urgent public health priority.

## Additional files

Additional file 1: Demographic characteristics of households receiving assistance from West Cheshire Foodbank by rooflessness status, 2013. (DOCX 15 kb)

Additional file 2: Number of unique and total adult and child recipients of emergency food. (DOCX 17 kb)

Additional file 3: Number and composition of foodbank visits each year. (DOCX 15 kb)

Additional file 4: Results of bivariate negative binomial regression models exploring associations between income and multiple deprivation and number of visits to West Cheshire Foodbank. (DOCX 12 kb)
