# Supplementary material for: Please sir, I want some more: an exploration of repeat foodbank use
Source: BMC Public Health. 2017 Nov 21;17:828. doi: 10.1186/s12889-017-4847-x (PMC5697111; doi:10.1186/s12889-017-4847-x)
Supplement: Supplementary file 1 — Demographic characteristics of households receiving assistance from West Cheshire Foodbank by rooflessness status, 2013. (DOCX 15 kb) [file 12889_2017_4847_MOESM1_ESM.docx]

Additional File 1: Demographic characteristics of households receiving assistance from West Cheshire Foodbank by rooflessness status, 2013. Chart. Word document

|  |  | **Housed households** | | | **Roofless households** | | |
| --- | --- | --- | --- | --- | --- | --- | --- |
|  |  | **Number of households (*n*)** | **Proportion of households (%)** | **Mean annual visits** | **Number of households (*n*)** | **Proportion of households (%)** | **Mean annual visits** |
| **Age group** | 17-24 | 284 | 13.9 | 2.6 | 16 | 17.8 | 1.9 |
|  | 25-64 | 1,709 | 83.9 | 3.6 | 74 | 82.2 | 4.0 |
|  | 65 and older | 45 | 2.2 | 2.9 | 0 | 0.0 | - |
| **Household type** | One person | 1,230 | 60.2 | 3.7 | 85 | 94.4 | 3.7 |
|  | Couple, no children | 187 | 9.2 | 2.9 | 2 | 2.2 | 2.0 |
|  | Couple parent with children | 360 | 17.6 | 3.1 | 3 | 3.3 | 1.3 |
|  | Lone parent with children | 224 | 11.0 | 2.8 | 0 | 0.0 | - |
|  | Other family type | 41 | 2.0 | 2.5 | 0 | 0.0 | - |
| **Reason for referral** | Benefit change | 384 | 18.8 | 3.3 | 3 | 3.3 | 4.3 |
|  | Benefit delay | 785 | 38.4 | 3.7 | 42 | 46.7 | 4.0 |
|  | Debt | 187 | 9.2 | 3.4 | 4 | 4.4 | 1.8 |
|  | Domestic abuse | 23 | 1.1 | 2.2 | 0 | 0.0 | - |
|  | Low income | 300 | 14.7 | 3.4 | 5 | 5.6 | 5.4 |
|  | Sickness/ill health | 28 | 1.4 | 3.9 | 0 | 0.0 | - |
|  | Unemployed | 30 | 1.5 | 2.4 | 0 | 0.0 | - |
|  | ‘Other’ reason | 305 | 14.9 | 3.1 | 36 | 40.0 | 3.1 |
| **Total** | | 2,042 | 95.8 | 3.4 | 90 | 4.2 | 3.6 |
