# Supplementary material for: Please sir, I want some more: an exploration of repeat foodbank use
Source: BMC Public Health. 2017 Nov 21;17:828. doi: 10.1186/s12889-017-4847-x (PMC5697111; doi:10.1186/s12889-017-4847-x)
Supplement: Supplementary file 2 — Number of unique and total adult and child recipients of emergency food. (DOCX 17 kb) [file 12889_2017_4847_MOESM2_ESM.docx]

Additional file 2: Number of unique and total adult and child recipients of emergency food. Chart. Word document
