# Supplementary material for: Please sir, I want some more: an exploration of repeat foodbank use
Source: BMC Public Health. 2017 Nov 21;17:828. doi: 10.1186/s12889-017-4847-x (PMC5697111; doi:10.1186/s12889-017-4847-x)
Supplement: Supplementary file 3 — Number and composition of foodbank visits each year. (DOCX 15 kb) [file 12889_2017_4847_MOESM3_ESM.docx]

Additional file 3: Number and composition of foodbank visits each year. Chart. Word document
