# Supplementary material for: Please sir, I want some more: an exploration of repeat foodbank use
Source: BMC Public Health. 2017 Nov 21;17:828. doi: 10.1186/s12889-017-4847-x (PMC5697111; doi:10.1186/s12889-017-4847-x)
Supplement: Supplementary file 4 — Results of bivariate negative binomial regression models exploring associations between income and multiple deprivation and number of visits to West Cheshire Foodbank. (DOCX 12 kb) [file 12889_2017_4847_MOESM4_ESM.docx]

Additional file 4: Results of bivariate negative binomial regression models exploring associations between income and multiple deprivation and number of visits to West Cheshire Foodbank. Table. Word document

|  |  | **Unadjusted bivariate IRR** |
| --- | --- | --- |
| **Index of Multiple Deprivation quintile** | 1 (most deprived) | 1.000  (0) |
|  | 2 | 1.040  (0.047) |
|  | 3 | 1.065  (0.063) |
|  | 4 | 0.918  (0.057) |
|  | 5 (least deprived) | 1.148  (0.097) |
| **Income Deprivation quintile** | 1 (most deprived) | 1.000  (0) |
|  | 2 | 1.120*  (0.051) |
|  | 3 | 0.868  (0.054) |
|  | 4 | 0.981  (0.057) |
|  | 5 (least deprived) | 1.046  (0.078) |

IRR= Incidence rate ratio. Standard errors in parentheses.

* p<.05 ** p<.01 *** p<.001
